# The associations between physical-test performance and match performance in women’s Rugby Sevens players

**DOI:** 10.5114/biolsport.2023.119985

**Published:** 2022-11-18

**Authors:** Francesco S. Sella, William G. Hopkins, Christopher M. Beaven, Daniel T. McMaster, Nicholas D. Gill, Kim Hébert-Losier

**Affiliations:** 1 Te Huataki Waiora School of Health, University of Waikato Adams Centre for High Performance, Mount Maunganui, 3116, New Zealand; 2 Institute for Health and Sport, Victoria University, Melbourne, VIC 8001, Australia; 3 New Zealand Rugby, Wellington, 6011, New Zealand

**Keywords:** Female athletes, Team sports, Training, Match actions, Match running

## Abstract

Evaluating the relationships between physical-test and match performance in team sports could be useful for training prescription and athlete evaluation. Here we investigated these relationships in women’s Rugby Sevens. Thirty provincial-representative players performed Bronco-fitness, countermovement-jump, acceleration, speed, and strength tests within two weeks before a two-day tournament. Match-running and match-action performance measures were provided by GPS units and video analysis. Generalised and general linear mixed models were employed to estimate the effect of a two standard-deviation difference in physical-test measures on match measures. Effect magnitudes were assessed via standardisation (using the between-player SD) and, for effects on tries scored, also via match winning (based on simulating matches). Evidence for substantial and trivial true magnitudes was provided by one-sided interval-hypothesis tests and Bayesian analysis. There was good evidence of positive effects of many physical-test measures on match high-intensity running, with large effects for jump height and acceleration. There was some evidence of small-moderate positive effects of speed and Bronco, and of small-moderate negative effects of maximal strength and jump height, on match total running and high intensity changes in speed. The evidence was generally inadequate for associations between physical-test measures and match actions, but there was good evidence of small-large positive effects of back squat and jump height on tries scored. Enhancing players’ jump height and back-squat performance might therefore increase the likelihood of match success in women’s Rugby Sevens.

## INTRODUCTION

Women’s Rugby Sevens is an intermittent, field-based team sport characterised by high-intensity activities and collisions [1]. Two teams of seven players contest matches over two 7-min halves in a tournament format over 2–3 days. Similar to other team sports, a combination of technical, tactical, and physical factors determines success in women’s Rugby Sevens [2]. While some of these factors (e.g., tactical awareness, decision making, and passing accuracy) are independent of physical measures, evaluating the relationships between physical-test and match performance could provide helpful information to implement specific training programs for enhancing match performance [2] and refining athlete evaluation.

In a previous study on women’s Rugby Sevens across different playing levels, performance in various physical tests (10-m acceleration, 40-m sprint, Yo-Yo Intermittent Recovery Test Level 1, vertical jump) had moderate to large correlations with some match-running activities, including total distance, distance covered >5 m∙s^−1^, and maximal speed [3]. However, several other measures of match running from the women’s Rugby Sevens literature such as number of sprints and accelerations [4, 5] were not included in this study; match actions were also not included. In provincial-representative and international-level men’s Rugby Sevens players, moderate to large correlations were observed between numerous physical-test measures and various match actions (e.g., tries scored) [6], but no studies have tested the relationships between physical-test measures and match actions in women’s Rugby Sevens. Therefore, in this study, we explored the associations of a range of physical-test measures with various match-running and match-action measures in women’s Rugby Sevens players.

## MATERIALS AND METHODS

### Subjects

Thirty women’s Rugby Sevens players (age: 22 ± 5 y, height: 1.68 ± 0.05 m, mass: 69 ± 7 kg) representing five different New Zealand Provincial Union teams participated in the study. Each participant provided written informed consent and ethical approval was granted from the University of Waikato research ethics committee.

### Study design

The association between physical-test performance and match performance in women’s Rugby Sevens players was examined using a descriptive correlation design. Participants performed a battery of physical tests within the two weeks before a two-day tournament. Match-running and match-action data were collected as measures of athlete performance using GPS units and video analysis. All tests employed are commonly used in Rugby [6–8].

### Acceleration and maximal speed

Acceleration and maximal speed abilities were assessed over a 40-m sprint on an outdoor artificial turf. Single beam timing lights (Brower Timing System, Utah, USA) were positioned at 0, 10, 30, and 40 m. The first gate was set at a 0.5 m height, while the remaining were set at 0.75 m. Prior to performing the sprints, participants completed a 10-minute standardised warm up comprising of jogging, dynamic stretches, running drills, and three stride-outs at increasing intensity. Participants started each sprint in a standing split position 0.5 m behind the first gate and were instructed to “run as fast as possible” past the last gate. Each participant performed two maximal effort sprints, separated by three minutes of passive rest. Sprint time was measured to the nearest 0.01 second. The fastest 10-m, 30-m, 40-m, and 30–40 m times were transformed into average running speeds and used for analysis. The speed over 10 m was used as an indicator of acceleration ability, while 30-m, 40-m, and 30–40 m speeds were used as measures of maximal speed abilities. The re-test reliability (intraclass correlation coefficient, ICC) for 10-m, 30-m, and 40-m sprint times in women’s Rugby Sevens players using similar equipment was 0.90, 0.95, and 0.96 [8].

### Fitness

The 1.2 km shuttle run test, also known as the Bronco test [7], was used as a measure of fitness. The test was performed outdoors, on artificial turf, in running shoes. The protocol consists of a continuous 20, 40, 60 m straight shuttle run, completed five times at maximal intensity (i.e., 20 m and back, 40 m and back, 60 m and back) [9]. Total running time was recorded with a stopwatch. Average running speed was calculated from total time and used for analysis. The ICC for Bronco time was 0.99 in men’s and women’s team-sport players combined [10].

### Countermovement jump

Bodyweight countermovement jumps were assessed using dualaxis force plates (PASPORT force plate, PASCO, California, USA) and analysed using custom-made software (Weightroom, HPSNZ, Auckland, New Zealand) sampling at 100 Hz. Participants started from an upright standing position with their hands-on hips and were instructed to “bend their knees to a self-selected depth and to jump as high as possible”. Two warm up trials were given, followed by two sets of three jumps separated by two minutes rest. The best jump height (calculated from flight time) recorded by each athlete was included in the analysis. The re-test reliability of the best jump height out of three countermovement jumps was 0.97 (ICC) and 3.6% (typical error) in women’s soccer players [11].

### Maximal strength

Back squat and bench press exercises were used to evaluate lower-body and upper-body maximal strength. For the back squat, athletes started from a standing position and were instructed to “lower until the thighs are parallel to the floor and then come up in the starting position”. A miss was recorded if participants failed to meet the proper depth or successfully come up in a straight position. For the bench press, athletes started with the arms fully straight, and were instructed to “lower the bar to the chest and press all the way up” while keeping the glutes in contact with the bench. If an athlete bounced the bar on the chest or failed to press the bar all the way up to a fully extended arms position, a fail was given. Each participant completed two warm up sets at sub-maximal intensities. Thereafter, participants were given three attempts to reach their 2–3 repetition maximum (RM) in each lift, with three minutes rest between attempts. One repetition maximum (1RM) from the lifts was calculated using the formula of Mayhew et al. [12]. ICCs for 1RM testing were ≥0.97 in women’s team-sport players [13].

### Match performance

Match-running and match-action data were collected during the New Zealand National Rugby Sevens tournament, a two-day tournament between the New Zealand Provincial Unions where each team competes in 5 to 6 matches. Match data were considered for players that completed at least a full 7-minute half of a match; therefore, resulting in 1 to 6 files for each player and a total of 119 files included in the analysis (6 players = 1 match, 2 players = 2 matches, 2 players = 3 matches, 5 players = 4 matches, 7 players = 5 matches, 8 players = 6 matches).

### Match running

Match running was measured using GPS units (VX Sport 220, Visuallex Sport International, Wellington, New Zealand) sampling at 10 Hz. The validity and reliability of devices with a similar sampling rate have been investigated previously [14, 15]. Each athlete wore the same GPS unit in every match in a fitted vest under the playing jersey. Data were downloaded and analysed post-tournament using the manufacturer’s software (VX View software, Sport International, Wellington, New Zealand). Match files were trimmed to include only the time players were on the field. The variables analysed were based on women’s Rugby Sevens research [3, 4, 16] and were described as the frequency of efforts or cumulative distance covered in different speed zones (see Table 1). Sprints were defined as running efforts that required an increase of ≥0.70 m∙s^−1^ within a second and that reached ≥2.8 m∙s^−1^ [17]. High-intensity accelerations and decelerations represented the the total number of accelerations and decelerations performed ≥2.0 m∙s^−2^.

**TABLE 1:** Match-performance variables predicted from mixed models without a physical-test predictor.

	Mean^a^	Standard deviations^b^		
		Within-player	Between-player	Observed in matches
**Match running**				
Match maximal speed (m∙s^−1^)	7.4	±8%	±6%	±10%
Distance > 7.5 m∙s^−1^ (m)	2	×/÷ 3.8	×/÷ 1.96	×/÷ 4.4
Distance > 5.5 m∙s^−1^ (m)	76	×/÷ 1.55	×/÷ 1.36	×/÷ 1.71
Distance > 5.0 m∙s^−1^ (m)	113	×/÷ 1.42	±23%	×/÷ 1.50
Distance > 4.7 m∙s^−1^ (m)	145	×/÷ 1.34	±18%	×/÷ 1.40
Distance 5.0–7.5 m∙s^−1^ (m)	108	×/÷ 1.41	±20%	×/÷ 1.48
Distance > 3.5 m∙s^−1^ (m)	347	±18%	±15%	±24%
Distance 3.5–5.0 m∙s^−1^ (m)	233	±22%	±23%	×/÷ 1.33
Total distance (m)	1123	±8%	±7%	±11%
Sprints	28	±13%	±9%	±16%
Accelerations	40	±13%	±12%	±18%
Decelerations	19	±22%	±13%	±26%
High-intensity accelerations	34	±15%	±15%	±21%
High-intensity decelerations	13	±25%	±18%	×/÷ 1.32

**Match actions**				
Tries	0.30	×/÷ 4.7	×/÷ 1.89	×/÷ 5.3
Line breaks	0.50	×/÷ 3.0	×/÷ 2.1	×/÷ 3.8
Work rate	4.91	×/÷ 1.53	×/÷ 1.34	×/÷ 1.68
Carries	0.85	×/÷ 3.0	×/÷ 1.52	×/÷ 3.2
Tackle breaks	0.40	×/÷ 3.9	×/÷ 2.6	×/÷ 5.3
Effective attacking rucks	0.25	×/÷ 5.6	×/÷ 1.52	×/÷ 5.9
Handling errors	0.38	×/÷ 5.7	±0%	×/÷ 5.7
Tackles	2.00	×/÷ 1.84	×/÷ 1.34	×/÷ 2.0
Missed tackles	0.85	×/÷ 2.9	±23%	×/÷ 3.0
Turnovers won	0.30	×/÷ 5.3	×/÷ 1.77	×/÷ 5.9

a Uncertainty (×/÷90% CL): for match running, 1.02–1.41; for match actions, 1.12–1.45.

b Derived from the mixed model: within-player is the residual, between-player is from the player identity, and the observed is their combination (via variances). Values ≥30% are shown as factors. Uncertainty (×/÷90% CL): for match running, 1.01–1.43; for match actions, 1.06–1.94.

### Match actions

The first author coded match actions using video analysis. The match actions included in the analysis and their operational definitions are presented in Table 2. These measures were chosen to represent different areas of the game in agreement with previous Rugby Sevens studies [6, 18]. Intra-rater reliability for the analysis was evaluated by re-analysing 10 random matches four weeks apart and calculating the percentage error, as described by Hughes et al. [19]. Errors observed for all match activities were within a 5% error limit, which was deemed acceptable.

**TABLE 2:** Operational definitions of match actions included in the analysis.

Match action	Description
**Attack**	
Tries	Count of tries scored by the player
Line breaks	Count of times the ball carrier breaks the defensive line
Carries	Count of times a player carries the ball into contact
Tackle breaks	Count of tackles evaded by the ball carrier
Effective attacking rucks	Count of attacking rucks in which the player successfully clears the opposition making the ball available to play
Handling errors	Sum of knock-ons, passes to ground, and dropped balls by the player
**Defence**	
Tackles	Count of tackles completed by the player
Missed tackles	Count of tackles attempted and missed by the player
Turnovers won	Count of times a player turns over the ball into an offensive situation from a defensive play
**Combined**	
Work rate	Sum of tries, line breaks, carries, tackle breaks, effective attacking rucks, tackles, and turnovers won

### Statistical Analysis

Data were analysed with the Statistical Analysis System (University Edition of SAS Studio, version 9.4, SAS Institute, Cary NC). Pearson correlations between each pair of physical-test variables were derived as a correlation matrix, and the variables were ordered to reveal clusters of similar variables (higher correlations within clusters than between clusters). The same analyses were performed for matchperformance variables.

For measures of match performance that were counts or proportional to counts, the association between each physical characteristic and the measure was analysed with Poisson regression using the generalised linear mixed model procedure (Proc Glimmix) with a log link. This procedure allows modelling of count variables and accounting for repeated match-performance measurements on the same player. Each physical characteristic (predictor) presented in Table 3 was entered in the model separately as a numeric linear fixed effect to allow estimation of the effect of a two standard-deviation (2-SD) difference in the predictor on match performance [6, 20]. The number of the match played by each athlete in the tournament, and the log of total match time (as a fraction of a 14-min match) for each player in each match, were included as numeric linear fixed effects to estimate the tournament trend of the dependent variable and to adjust each player’s score to a mean match time, respectively. Random effects in the model were nominal variables representing player identity (to adjust for between-player differences in means), match identity (to adjust for between-match differences in means), and team identity (to adjust for between-team differences in means). Over- or under-dispersion of the residual variance was estimated, which was particularly important for measures representing counts of running bouts. Distance covered >7.5 m∙s^−1^ produced an unrealistic estimate of over-dispersion, so it was analysed with the general linear mixed-model procedure (Proc Mixed) after log transformation, with values of 0 first set to half of the smallest non-zero value. The fixed and random effects were the same as for the Poisson-regression model. The same general linear mixed model was used to analyse the only measure of match performance that was not a count or count of bouts (maximal speed), which was also log transformed. Estimates for the tournament trend were also obtained from the generalised and the general linear mixed models without a physical-test predictor.

**TABLE 3:** Characteristics of the players in the physical tests. Data are mean^a^ ± SD^b^ (sample size).

10-m average speed (m·s^−1^)	5.30±0.18 (16)
30-m average speed (m·s^−1^)	6.47±0.23 (16)
40-m average speed (m·s^−1^)	6.72±0.28 (16)
Maximal speed (m·s^−1^)	7.66±0.49 (16)
Bronco average speed (m·s^−1^)	3.51±0.27 (16)
Bench press 1RM (kg)	59±10 (28)
Back squat 1RM (kg)	90±15 (26)
CMJ height (cm)	32.2±4.1 (20)

CMJ = Countermovement jump, 1RM = One repetition maximum.

a Uncertainty (±90% CL): 1.5–5.7%.

b Uncertainty (×/÷90% CL): 1.26–1.36.

The qualitative magnitude of the effects was assessed using standardisation, with threshold values for small, moderate, large, very large, and extremely large calculated as 0.2, 0.6, 1.2, 2.0, and 4.0 of the observed between-player SD [20], derived by combining the variances represented by player identity (true differences between players) and the residual (within-player between match variance), and adjusted for small samples [21]. Effect magnitudes for tries were also determined as the factors associated with an increase in the number of tries scored by a team to give the team 1, 3, 5, 7, and 9 extra wins every 10 matches, representing small, moderate, large, very large, and extremely large effects [20]. The factors were estimated using a simulation based on points scored by all the teams in the tournament. There was a mean of 2.7 tries scored per team per match (5 points per try), with a 52% probability of converting a try (2 points per conversion). There were no field goals or penalties. We assumed a team had 10 try-scoring opportunities in a match on the basis of our experience (The resulting magnitude thresholds were not sensitive to the number of opportunities). An opponent team was assumed to have an unchanging probability of scoring a try per opportunity equal to 27% (2.7 tries per 10 opportunities). The factor associated with a given increase in wins per 10 matches allowed for the affected team to have a try-scoring probability per trial <27% before the factor was applied (probability = 0.27/√factor), but it increased to >27% after the factor was applied (probability = 0.27*√factor). Simulations were performed to generate scores in 10,000 matches for the two teams before and after the factor was applied, then wins were scored as 1 and loss or draw as 0. The factors giving 1, 3, 5, 7, and 9 extra wins every 10 matches were found by “trial and error”. Corresponding magnitude thresholds for factor increase/decrease were 1.20/0.83, 1.75/0.57, 2.60/0.38, 4.10/0.24, and 11.0/0.09. The spreadsheet of simulations is available on request.

Physical-test scores are shown as means and standard deviations (SDs). Means of the dependent variables are shown as the back-transformed least-squares means with SDs in back-transformed ± percent units (when <30%) or × /÷ factor units (when >30%) derived from a model without a physical-test predictor. Effects are presented in percent units with uncertainty expressed as ± 90% compatibility (or confidence) limits (CL), when either the effect or the ± 90% CL were <30%; otherwise, factor effects with × /÷90% CL are reported. Decisions about magnitudes accounting for sampling uncertainty were based on one-sided interval hypothesis tests, according to which a hypothesis of a given magnitude (substantial, non-substantial) is rejected if the 90% compatibility interval falls outside that magnitude [22, 23]. P-values for the tests were therefore the areas of the sampling t-distribution of the effect falling in the hypothesised magnitude, with the distribution centred on the observed effect. Hypotheses of inferiority (substantial negative) and superiority (substantial positive) were rejected if their respective p-values (p_−_ and p_+_) were <0.05; rejection of both hypotheses represents a decisively trivial effect in equivalence testing. The hypothesis of non-inferiority (non-substantial-negative) or non-superiority (non-substantial-positive) was rejected if its p-value (p_N–_ = 1 – p_−_ or p_N+_ = 1 – p_+_) was <0.05, representing a decisively substantial effect in minimal-effects testing. A complementary Bayesian interpretation of sampling uncertainty was also provided, when at least one substantial hypotheses was rejected: the p-value for the other hypothesis is the posterior probability of a substantial true magnitude of the effect in a Bayesian analysis with a non-informative prior [22–24], and it was interpreted qualitatively using the following scale: >0.25, possibly; >0.75, likely; >0.95, very likely; and >0.995, most likely [20]. The probability of a trivial true magnitude (1 – p_−_–p_+_) was also interpreted with the same scale. Possible or likely magnitudes are categorised as some evidence for those magnitudes; very likely and most likely are categorised as good evidence. Probabilities were not interpreted for unclear effects: those with inadequate precision at the 90% level, defined by failure to reject both substantial hypotheses (p_−_ >0.05 and p_+_ >0.05). Effects on magnitudes and probabilities of a weakly informative normally distributed prior centred on the nil effect and excluding extremely large effects at the 90% level were also investigated [24, 25].

Effects with adequate precision at the 99% level (p_−_<0.005 or p_+_<0.005) are highlighted in bold in tables, since these represent stronger evidence against substantial hypotheses than the 90% level and therefore incur lower inflation of error with multiple hypothesis tests. For practitioners considering implementation of a treatment based on an effect in this study (e.g., training to improve try scoring by increasing jump height), the effect needs only a modest chance of benefit (at least possibly increased try scoring, p_+_>0.25) but a low risk of harm (most unlikely impaired try scoring, p_−_<0.005). Substantial effects highlighted in bold therefore represent potentially implementable effects. However, it is only for effects on tries scored assessed via match winning that the outcomes have direct relevance to benefit and harm (winning and losing matches); these effects were also deemed potentially implementable when the chance of benefit outweighed an otherwise unacceptable risk of harm (the odds ratio of benefit to harm >66.3) [26]. For these effects, the potential for benefit and harm was also investigated for realistic changes in physical-test measures (less than 2 SD).

## RESULTS

### Physical tests and match performance

Mean values and between-subjects SD of physical-test scores are presented in Table 3, while means of the dependent variables with the within-player, between-player, and observed SD are reported in Table 1. The within-player SD represents the match-to-match within-player variation, the between-player SD is the true difference between players, and the observed SD is the combination of the within- and between-player SDs representing the observed between-player SD in a typical match.

### Correlations within and between groups of variables

Correlation matrices for the physical-test and match measures are shown in the Supplementary Tables 1–4, where clusters of variables have been identified as those with correlations ≥0.50 within clusters and <0.50 between clusters. The clusters of variables in each correlation matrix are delineated in the Supplementary Table 4, because correlated variables were expected to have similar effects. There were two overlapping clusters of physical-test variables for maximal-speed running measures, one with 10-m speed and one with Bronco; there was also a well-defined cluster for strength measures (Supplementary Table 1). The correlation matrix for measures of match running revealed four clusters, representing running at high intensities, running at lower intensities (with distance >5.5 m∙s^−1^ in both clusters), total running (with sprinting, accelerations, and decelerations contributing to this concept), and high-intensity changes in speed (Supplementary Table 2). Fewer and less well-defined clusters of variables were found for match actions, with a cluster for tries and line breaks, and a cluster (with one correlation of 0.47) for work rate, carries, and tackle breaks (Supplementary Table 3). No clusters contained match-running and match-action variables, but correlations of match running with match actions (Supplementary Table 4) revealed similar magnitudes within the running and action clusters; in particular, tries and line breaks had negative correlations with measures of total running and positive correlations with high-intensity running.

### Effects of physical-test scores on match performance

The effects of a 2-SD difference in physical-test scores on match running and match actions are presented in Table 4. Compatibility intervals and Bayesian probabilities are shown for a minimally informative prior, since appreciable shrinkage occurred with the weakly informative prior for only one effect, jump height on tries scored. In this instance, the factor effect reduced to 3.1, 90% compatibility limits × /÷2.6, but the magnitude remained large, very likely substantial, and potentially implementable with the odds-ratio assessment of benefit and harm defined by thresholds for match winning.

**TABLE 4:** Effects of a 2-SD difference in physical characteristics on match running and match actions. Data are percent effects with ± 90% compatibility limits, or factor effects with×/÷90% compatibility limits; the observed magnitude and probability of a substantial true effect are also shown. Horizontal and vertical dashed and solid lines divide the match and physical-test measures into clusters defined by the correlations between measures within and between clusters shown in the Supplementary Tables 1–3.

	10-m speed (m·s^−1^)	40-m speed (m·s^−1^)	Bronco average speed (m·s^−1^)	Bench press 1RM (kg)	Back squat 1RM (kg)	CMJ height (cm)
**Match running**						

Match maximal speed	12.5,±7.1%;	7.4,±6.6%;	8.0,±6.9%;	3.0,±4.8%;	6.1,±4.8%;	13.6,±5.9%;
	**Large** ↑***	Moderate ↑**	Moderate ↑**	Small ↑*^0^	**Moderate** ↑**	**Large** ↑****
Distance >7.5 m·s^−1^	2.6,×/÷2.4;	2.0,×/÷2.3;	2.4,×/÷2.4;	1.28,×/÷1.90;	1.41,×/÷1.91;	3.5,×/÷2.2;
	Moderate ↑**	Small ↑**	Small ↑**	Trivial	Small ↑	**Moderate** ↑***

Distance >5.5 m·s^−1^	1.59,×/÷1.50;	1.56,×/÷1.38;	1.69,×/÷1.35;	1.34,×/÷1.26;	1.44,×/÷1.26;	1.95,×/÷1.22;
	Moderate ↑**	**Moderate** ↑**	**Moderate** ↑***	**Small** ↑**	**Moderate** ↑***	**Large** ↑****

Distance >5.0 m·s^−1^	1.42,×/÷1.35;	1.40,×/÷1.26;	1.50,×/÷1.24;	29,±23%;	1.31,×/÷1.19;	1.63,×/÷1.18;
	Moderate ↑**	**Moderate** ↑***	**Moderate** ↑***	**Moderate** ↑**	**Moderate** ↑***	**Large** ↑****
Distance >4.7 m·s^1^	1.37,×/÷1.27;	1.33,×/÷1.19;	1.39,×/÷1.19;	17,±18%;	17,±19%;	1.48,×/÷1.16;
	Moderate ↑**	**Moderate** ↑***	**Moderate** ↑***	Small ↑**	Small ↑**	**Large** ↑****
Distance 5.0–7.5 m·s^−1^	1.34,×/÷1.32;	1.34,×/÷1.23;	1.44,×/÷1.22;	29,±21%;	1.31,×/÷1.18;	1.58,×/÷1.18;
	Moderate ↑**	**Moderate** ↑**	**Moderate** ↑***	**Moderate** ↑***	**Moderate** ↑***	**Large** ↑****

Distance >3.5 m·s^−1^	10,±16%;	11,±11%;	12,±11%;	-8,±10%;	↓	2,±12%;
	Small ↑	Small ↑**	Small ↑**	Small ↓*^0^		Trivial
Distance 3.5–5.0 m·s^−1^	-1,±23%;	1,±20%;	-1,±19%;	-18,±12%;	-19,±13%;	-19,±16%;
	Trivial	Trivial	Trivial	**Moderate** ↓**	**Moderate** ↓***	Moderate ↓**
Total distance	3.4,±9.2%;	6.4,±7.1%;	9.2,±6.5%;	-5.5,±4.8%;	-4.5,±5.2%;	-1.1,±7.1%;
	Small ↑	Small ↑**	**Moderate** ↑***	Small ↓**	Small ↓**	Trivial
Sprints	3.3,±9.3%;	6.1,±6.7%;	7.0,±6.3%;	-8.9,±6.3%;	-7.4,±7.2%;	-2.1,±6.8%;
	Small ↑	Small ↑**	Small ↑**	**Moderate** ↓**	Small ↓**	Trivial
Accelerations	-2,±12%;	4,±11%;	10,±12%;	-5.7,±8.1%;	-4.9,±8.9%;	3,±11%;
	Trivial	Small ↑	Small ↑**	Small ↓*^0^	Small ↓	Trivial
Decelerations	9,±15%;	10,±10%;	14.6,±9.2%;	-5,±11%;	-3,±12%;	-1,±14%;
	Small ↑	Small ↑**	**Moderate** ↑***	Small ↓	Trivial	Trivial

High-intensity accelerations	13,±19%;	9,±15%;	11,±14%;	-12.3,±9.0%;	-10,±10%;	5,±15%;
	Small ↑	Small ↑	Small ↑**	**Moderate** ↓**	Small ↓**	Small ↑
High-intensity decelerations	9,±21%;	8,±16%;	21,±12%;	-2,±15%;	6,±16%;	6,±18%;
	Small ↑	Small ↑	**Moderate** ↑***	Trivial	Small ↑	Small ↑

**Match actions**						

Tries	1.3,×/÷2.8;	1.4,×/÷2.3;	1.5,×/÷2.3;	1.55,×/÷1.92;	2.5,×/÷2.0;	3.8,×/÷2.8;
via standardisation	Trivial	Trivial	Trivial	Small ↑*^0^	**Small** ↑**	Moderate ↑**
via match winning	Small ↑	Small ↑	Small ↑	Small ↑	Moderate ↑***	Large ↑***
Line breaks	2.6,×/÷2.7;	1.2,×/÷2.1;	1.0,×/÷2.2;	1.37,×/÷1.86;	2.1,×/÷1.9;	2.8,×/÷2.3;
	Moderate ↑**	Trivial	Trivial	Small ↑	Small ↑**	Moderate ↑**

Work rate	0.99,×/÷1.38;	1.21,×/÷1.30;	1.31,×/÷1.28;	16,±29%	1.37,×/÷1.28;	20,±29%
	Trivial	Small ↑*^0^	Small ↑**;	Small ↑*^0^	Moderate ↑**	Small ↑*^0^
Carries	1.12,×/÷1.93;	1.22,×/÷1.72;	170,×/÷1.71;	1.10,×/÷1.59;	1.44,×/÷1.61;	1.47,×/÷1.66;
	Trivial	Trivial	Small ↑**	Trivial	Small ↑*^0^	Small ↑*^0^
Tackle breaks	0.7,×/÷2.6;	1.4,×/÷2.8;	1.4,×/÷2.7;	1.0,×/÷2.2;	1.7,×/÷2.3;	2.9,×/÷3.1;
	Small ↓	Small ↑	Trivial	Trivial	Small ↑*^0^	Small ↑**

Effective attacking rucks	0.4, × /÷4.7;	0.5, × /÷4.5;	1.2, × /÷4.4;	1.39, × /÷1.80;	1.19, × /÷1.90;	1.1, × /÷2.2;
	Small ↓	Small ↓	Trivial	Trivial ↑^0^*	Trivial	Trivial

Handling errors	1.3, × /÷2.4;	1.5, × /÷2.1;	1.8, × /÷2.1;	1.03, × /÷1.73;	1.20, × /÷1.76;	1.73, × /÷1.92;
	Trivial	Small ↑*^0^	Small ↑*^0^	Trivial	Trivial	Small ↑*^0^

Tackles	0.88, × /÷1.60;	1.21, × /÷1.52;	1.30, × /÷1.56;	1.19, × /÷1.33;	1.29, × /÷1.34;	0.86, × /÷1.44;
	Trivial	Small ↑	Small ↑	Small ↑*^0^	Small ↑*	Small ↓

Missed tackles	0.90, × /÷1.75;	0.97, × /÷1.60;	1.08, × /÷1.68;	1.21, × /÷1.43;	1.15, × /÷1.46;	1.04, × /÷1.55;
	Trivial	Trivial	Trivial	Trivial ↑^0^*	Trivial	Trivial

Turnovers won	1.4, × /÷2.2;	1.24, × /÷1.95;	1.6, × /÷2.0;	0.9, × /÷2.0;	0.6, × /÷2.0;	0.5, × /÷2.1;
	Trivial	Trivial	Small ↑*^0^	Trivial	Small ↓*^0^	Small ↓**

CMJ = Countermovement jump, 1RM = One repetition maximum. With the exception of tries, magnitudes are based on the following scale for standardised changes in the mean using the observed between-player SD (Table 1): <0.2, trivial; 0.2–0.6, small; 0.6–1.2, moderate; 1.2–2.0, large; 2.0–4.0, very large; >4.0 extremely large. Magnitude thresholds for tries are based on simulations of Rugby Sevens matches (see Methods). Reference-Bayesian likelihoods of substantial change: *possibly; **likely; ***very likely; ****most likely. *** and **** indicate rejection of the non-superiority or non-inferiority hypothesis (p_N–_ or p_N+_<0.05 and <0.005 respectively). ↑ and ↓ indicate a substantial positive and negative effect, respectively. Reference-Bayesian likelihoods of trivial change: ^0^possibly; ^00^likely; ^000^very likely, ^0000^most likely. Likelihoods are not shown for effects with inadequate precision at the 90% level (failure to reject any hypotheses: p>0.05). Magnitudes in **bold** have acceptable precision with 99% compatibility limits.

### Match running

Large positive effects were observed for jump height on match maximal speed and distance covered at high intensity, and for acceleration on match maximal speed, where the effects had sufficient precision for the true magnitudes to be very likely or most likely substantial. Acceleration, speed, Bronco, and strength scores displayed consistent moderate positive effects on distance covered at high intensity during matches, with some effects showing sufficient precision for the true magnitudes to be very likely substantial. Moderate positive effects where precision was sufficient for the true magnitude to be very likely substantial were also evident for 30-m speed on match maximal speed, for jump height on distance >7.5 m∙s^−1^, and for Bronco on match total distance, decelerations, and high-intensity decelerations.

Measures where the effects had adequate precision but were only likely substantial included small and moderate positive effects for speed, Bronco, and back squat on match maximal speed, and for speed and Bronco on match distance covered >7.5 m∙s^−1^ and on some measures of total running. In contrast, small to moderate negative effects were observed for strength and jump height on some variables contributing to total match running and high intensity changes in speed. Precision was consistently inadequate for speed on match distance 3.5–5.0 m∙s^−1^, accelerations, high-intensity accelerations, high-intensity decelerations, and for strength and jump height on match decelerations and high-intensity decelerations.

### Match actions

Moderate and small positive effects were observed for jump height and back squat on tries scored when assessed using standardisation, with adequate precision at the 90% and 99% levels respectively; both were likely substantial. Greater positive effects characterised the same predictors for tries scored when assessed via match winning, and the effects became very likely substantial; both effects had adequate precision only at the 90% level, hence the risk of harm was too high (p_−_>0.005) for a conservative assessment of implementability, but they were potentially implementable when considering the odds ratio of benefit to harm (3700 and 4200, respectively). For these predictors, changes as small as 0.25 SD predicted tries scored that were at least possibly beneficial and with negligible risk of harm, but changes of 0.2 SD were unlikely to be beneficial.

A number of predictors had small or moderate positive effects but were only likely or possibly substantial: back squat and jump height on line breaks, work rate, carries, tackle breaks; 10-m speed on line breaks; maximal speed on work rate; Bronco on work rate and carries; and strength on tackles. On the other hand, back squat and jump height displayed small negative effects on turnovers won with adequate precision, but the effect was only possibly or likely substantial. Precision was inadequate for several predictors on various match-action measures, including the observed small positive effects of measures of running speed and bench press on match winning. Changes in measures of running speed and in bench press smaller than 2 SD were either unclear or at least likely trivial.

### Tournament trend

When considering the effects obtained without a physical-test predictor, the measures of match total running and high-intensity changes in speed displayed small to moderate negative trends across the tournament, with adequate precision but only possibly or likely substantial magnitudes. The trend for the match measures of high-speed running ranged from small likely reductions through to small possible increases, but three of the six measures had inadequate precision. There was a similar pattern for match actions, with seven of the 11 measures lacking adequate precision. The tournament trends sometimes changed substantially with different physical-test predictors in the model, but overall the trends were similar to those without predictors.

## DISCUSSION

There was good evidence of positive effects of many physical-test measures on match high-intensity running in women’s Rugby Sevens players. Furthermore, there was some evidence of positive effects of speed and Bronco on match total running and high intensity changes in speed, and of negative effects of maximal strength and jump height on match total running and high intensity changes in speed. There were fewer substantial associations between physical-test measures and match actions: good evidence of positive effects of squat and jump height on tries via standardisation and match winning, and some evidence of positive effects of various predictors on some measures. Small positive effects of measures of running speed and bench press on match winning were observed, but these were unclear. The similarity of the effects observed for some predictors and/or dependent variables reflects the high correlations observed between variables.

Improving back squat and jump height performance could be advantageous for enhancing match winning in provincial-representative women’s Rugby Sevens, as the effects for these tests on tries scored were deemed potentially implementable. In Table 4 we have presented the effect of a 2-SD difference in these tests on match performance, representing the difference of moving from a typical low to a typical high value [20]. Achieving such a difference would be unrealistic for players already displaying high test values, but a change of 0.25 SD should be achievable for most players and was still potentially beneficial. There was less evidence for a beneficial effect (small but unclear) of measures of running speed and bench press on match winning; some changes smaller than 2 SD were unclear and therefore worth further investigation for potential benefit.

The magnitudes of the positive effects (small to large) for match-running measures align with moderate to large correlations (*r* = ~0.3–0.7) between performance in various physical-test and match-running measures reported in the only other comparable study of women’s Rugby Sevens players [3]. The authors in that study combined junior, senior, and professional levels, so the correlations within each level would therefore likely be lower. In a study of provincial-representative and international-level men’s Rugby Sevens players [6], some of the associations between physical-test measures and match actions were similar to ours in magnitude. A point of difference was that tries scored was moderately correlated with 10-m momentum (sprint velocity multiplied by body mass) and repeated-sprint ability, whereas in our study the measures of running speed in the physical tests had trivial (although unclear) effects on tries scored assessed via standardisation. If this difference is not due simply to sampling variation, then the explanation must reside in differences between either the style of matches or the physiology of male and female players. Unfortunately, jump height and lower-body strength were not measured in the study of men’s Rugby Sevens [6], and there have been no other studies of the effects of these test measures on tries or other match actions in Rugby Sevens.

The small to moderate negative effects of maximal strength and jump height on match measures of total running and high-intensity changes in speed contrast with the moderate to large positive effects of these physical-test measures on match winning. Total running and high-intensity changes in speed are apparently negative match performance indicators, as evidenced by moderately lower total distance and distance covered at 3.5–5.0 m∙s^−1^ during wins compared to losses in international women’s Rugby Sevens [5]. Similarly, in the current study we observed consistently negative correlations between measures of total running and tries scored (Supplementary Table 4).

For most match-running and match-action measures, there was some evidence of small to moderate negative trends over the tournament, likely a result of accumulated fatigue or muscle damage. In line with these findings, professional and state-representative women’s Rugby Sevens players displayed small to very large reductions in several match-running measures over a two-day tournament, with greater reductions in state-level players [27]. Both professional and state-representative players also reported a large decline in recovery perception, a large increase in perceived soreness, and had large increases in muscle damage (creatine kinase concentration) over the tournament. No information regarding the tournament trend of match actions in women’s Rugby Sevens has been reported in other studies.

Due to the fact the physical tests were undertaken within a 14-day period before competition, it is possible that different teams with different training and tapering could have different relationships. To the extent that some of the test measures might be measuring the same underlying construct, the correlations between the measures are similar to reliability correlations, and the measurement error is negligible (in terms of standardisation) only when the correlation is ~0.99 or greater [28]. On this basis, only the 30-m and 40-m speed are effectively the same measure (Supplementary Table 1), and only one needs to be measured. Measures with lower correlations could either be measuring identical constructs with substantial measurement error or they could be measuring somewhat different constructs. A parsimonious set of physical tests that assess constructs making independent contributions to match performance would be useful for practitioners, but multiple linear regression with a much larger sample size of players is needed to identify them. The small sample size precluded such an analysis. A similar analysis with more players and matches to predict tries scored with the other match-performance measures might identify a parsimonious set of match measures for predicting match performance of individual players. A greater sample size of players and matches is also required to get more evidence about the magnitude of the unclear effects observed in this study, especially those on match winning.

## CONCLUSIONS

Ours is the first study to reveal potentially useful relationships between physical-test measures and match performance in women’s Rugby Sevens players. In particular, enhancing players’ jump height and back-squat performance could increase the likelihood of match success in women’s Rugby Sevens. Valuable future research would include multiple linear regression and experimental studies investigating the effect of changes in physical-test measures on match performance to support the promising utility of these findings for enhancing performance in women’s Rugby Sevens.

## Supplementary Material

The associations between physical-test performance and match performance in women’s Rugby Sevens players
