# Supplementary material for: The associations between physical-test performance and match performance in women’s Rugby Sevens players
Source: Biol Sport. 2022 Nov 18;40(3):775–85. doi: 10.5114/biolsport.2023.119985 (PMC10286606; doi:10.5114/biolsport.2023.119985)
Supplement: The associations between physical-test performance and match performance in women’s Rugby Sevens players [file JBS-40-119985-s1.pdf]

SUPPLEMENTARY TABLES

SUPPLEMENTARY TABLE 1. Correlations between physical tests mean values. The variables have been ordered and outlined to show clusters with generally higher correlations between variables within the clusters than between the clusters.

Variable	1	2	3	4	5	6	7	8
1. 10-m average speed		.70	.64	.51	.31	-.32	-.23	.05
2. 30-m average speed	.70		.99	.91	.59	-.27	-.37	.03
3. 40-m average speed	.64	.99		.96	.62	-.25	-.42	-.04
4. Maximal speed	.51	.91	.96		.62	-.19	-.46	-.13
5. Bronco average speed	.31	.59	.62	.62		.18	.09	.23
6. Bench press 1RM	-.32	-.27	-.25	-.19	.18		.76	.29
7. Back squat 1RM	-.23	-.37	-.42	-.46	.09	.76		.47
8. CMJ height	.05	.03	-.04	-.13	.23	.29	.47	

Uncertainty (90% compatibility limits): ~±0.31 to ~±0.03 for correlations of 0.00 to 0.95 respectively assuming a sample size of ~30.

SUPPLEMENTARY TABLE 2. Correlations between match running mean values. The variables have been ordered and outlined to show clusters with generally higher correlations between variables within the clusters than between the clusters.

Variable	1	2	3	4	5	6	7	8	9	10	11	12	13	14
1. Match maximal speed		.56	.61	.49	.40	.40	.06	-.25	.03	-.09	-.11	.03	-.07	.03
2. Distance >7.5 m·s^{-1}	.56		.53	.42	.32	.22	.08	-.19	.02	.05	-.03	.03	-.03	.00
3. Distance >5.5 m·s^{-1}	.61	.53		.94	.84	.88	.38	-.16	.17	-.01	.00	.24	.03	.30
4. Distance >5.0 m·s^{-1}	.49	.42	.94		.96	.98	.56	.01	.29	.15	.13	.37	.11	.45
5. Distance >4.7 m·s^{-1}	.40	.32	.84	.96		.96	.72	.23	.42	.28	.26	.52	.14	.52
6. Distance 5.0–7.5 m·s^{-1}	.40	.22	.88	.98	.96		.58	.05	.30	.15	.15	.39	.13	.48
7. Distance >3.5 m·s^{-1}	.06	.08	.38	.56	.72	.58		.83	.76	.64	.60	.73	.16	.46
8. Distance 3.5–5.0 m·s^{-1}	-.25	-.19	-.16	.01	.23	.05	.83		.72	.67	.63	.63	.12	.26
9. Total distance	.03	.02	.17	.29	.42	.30	.76	.72		.71	.70	.56	.23	.30
10. Sprints	-.09	.05	-.01	.15	.28	.15	.64	.67	.71		.85	.61	.41	.43
11. Accelerations	-.11	-.03	.00	.13	.26	.15	.60	.63	.70	.85		.56	.55	.46
12. Decelerations	.03	.03	.24	.37	.52	.39	.73	.63	.56	.61	.56		.14	.62
13. High-intensity accelerations	-.07	-.03	.03	.11	.14	.13	.16	.12	.23	.41	.55	.14		.69
14. High-intensity decelerations	.03	.00	.30	.45	.52	.48	.46	.26	.30	.43	.46	.62	.69	

Uncertainty (90% compatibility limits): ~±0.31 to ~±0.03 for correlations of 0.00 to 0.95 respectively assuming a sample size of ~30.

SUPPLEMENTARY TABLE 3. Correlations between match actions mean values. The variables have been ordered and outlined to show clusters with generally higher correlations between variables within the clusters than between the clusters.

Variable	1	2	3	4	5	6	7	8	9	10
1. Tries		.53	.33	-.05	.14	-.15	.00	-.10	-.17	.05
2. Line breaks	.53		.49	.08	.31	-.08	-.05	-.15	-.20	.13
3. Work rate	.33	.49		.54	.59	.12	.06	.41	-.15	.35
4. Carries	-.05	.08	.54		.47	-.06	-.07	-.12	-.08	.03
5. Tackle breaks	.14	.31	.59	.47		-.05	.01	-.17	-.07	.09
6. Effective attacking rucks	-.15	-.08	.12	-.06	-.05		.06	.06	.07	.00
7. Handling errors	.00	-.05	.06	-.07	.01	.06		.20	.23	-.09
8. Tackles	-.10	-.15	.41	-.12	-.17	.06	.20		.06	.06
9. Missed tackles	-.17	-.20	-.15	-.08	-.07	.07	.23	.06		-.19
10. Turnovers won	.05	.13	.35	.03	.09	.00	-.09	.06	-.19	

Uncertainty (90% compatibility limits): ~±0.31 to ~±0.03 for correlations of 0.00 to 0.95 respectively assuming a sample size of ~30.

SUPPLEMENTARY TABLE 4. Correlations between match running and match actions mean values. The variables have been ordered and outlined to show clusters with generally higher correlations between variables within the clusters than between the clusters.

Variable	Tries	Line breaks	Work rate	Carries	Tackle breaks	Effective attacking rucks	Handling errors	Tackles	Missed tackles	Turnovers won
Match maximal speed	.38	.31	.04	-.10	-.01	-.17	-.08	-.05	.00	-.14
Distance >7.5 $m \cdot s^{-1}$	.31	.17	.13	.09	-.03	-.03	-.02	.05	-.02	-.17
Distance >5.5 $m \cdot s^{-1}$	.32	.24	.15	-.06	-.02	-.06	-.13	.11	-.10	-.07
Distance >5.0 $m \cdot s^{-1}$	.26	.22	.17	-.06	-.04	-.05	-.08	.16	-.09	.03
Distance >4.7 $m \cdot s^{-1}$	.18	.16	.16	-.07	-.06	-.03	-.06	.20	-.07	.06
Distance 5.0–7.5 $m \cdot s^{-1}$	.21	.19	.16	-.08	-.03	-.05	-.08	.16	-.09	.07
Distance >3.5 $m \cdot s^{-1}$	-.08	-.05	.05	-.15	-.13	-.01	-.05	.29	.01	.16
Distance 3.5–5.0 $m \cdot s^{-1}$	-.27	-.21	-.05	-.15	-.13	.03	.00	.24	.08	.18
Total distance	-.10	-.11	.11	-.02	-.04	-.03	.02	.28	-.03	.12
Sprints	-.15	-.07	.21	.08	-.05	.07	.06	.39	.02	.06
Accelerations	-.18	-.06	.22	.08	-.02	.03	.00	.37	.02	.16
Decelerations	-.13	-.04	.01	-.17	-.18	.11	-.04	.26	-.03	.12
High-intensity accelerations	.00	.01	.23	.15	.03	.16	.01	.15	.00	.18
High-intensity decelerations	.01	.05	.20	.04	-.06	.12	.01	.21	-.08	.20

Uncertainty (90% compatibility limits): ~±0.31 to ~±0.03 for correlations of 0.00 to 0.95 respectively assuming a sample size of ~30.
